# Characterization of Brazilian Buriti oil biomaterial: the influence on the physical, chemical properties and behaviour of Goat Wharton’s jelly mesenchymal stem cells

**DOI:** 10.1590/1984-3143-AR2023-0071

**Published:** 2023-12-11

**Authors:** Camila Ernanda Sousa de Carvalho, Fernando da Silva Reis, Elis Rosélia Dutra de Freitas Siqueira Silva, Dayseanny de Oliveira Bezerra, Isnayra Kerolaynne Carneiro Pacheco, Ana Cristina Vasconcelos Fialho, José Milton Elias de Matos, Wanderson Gabriel Gomes de Melo, Yulla Klinger Pereira de Carvalho Leite, Napoleão Martins Argôlo, Maria Acelina Martins de Carvalho

**Affiliations:** 1 Programa de Pós-graduação em Zootecnia Tropical, Centro de Ciências Agrárias, Universidade Federal do Piauí, Teresina, PI, Brasil; 2 Programa de Pós-graduação em Ciências dos Materiais, Centro de Ciências da Natureza, Universidade Federal do Piauí, Teresina, PI, Brasil; 3 Programa de Pós-graduação em Tecnologias Aplicadas a Animais de Interesse Regional, Centro de Ciências Agrárias, Universidade Federal do Piauí, Teresina, PI, Brasil; 4 Programa de Pós-graduação em Odontologia, Centro de Ciências da Saúde, Universidade Federal do Piauí, Teresina, PI, Brasil; 5 Departamento de Patologia Clínica Dental, Centro de Ciências da Saúde, Universidade Federal do Piauí, Teresina, PI, Brasil; 6 Departamento de Morfologia, Centro de Ciências da Saúde, Universidade Federal do Piauí, Teresina, PI, Brasil

**Keywords:** cell growth, cytotoxicity, polyurethane

## Abstract

The Brazilian Buriti oil presents low extraction costs and relevant antioxidant properties. Thus, this work aimed to analyze Buriti oil biomaterial (BB), within its physicochemical properties, biocompatibility and cellular integration, with the purpose to the use as a growth matrix for Goat Wharton’s jelly mesenchymal stem cells. Biomaterials were produced from Buriti oil polymer (*Mauritia flexuosa*), for it’s characterization were performed Infrared Region Spectroscopy (FTIR) and Thermogravimetric Analysis (TG and DTG). The biointegration was analyzed by Scanning Electron Microscopy (SEM) and histological techniques. In order to investigate biocompatibility, MTT (3-(4,5-dimetil-2-tiazolil)-2,5-difenil-2H-tetrazólio) test and hemolytic activity tests were performed. The activation capacity of immune system cellswas measured by phagocytic capacity assay and nitric oxide synthesis . The BB presented an amorphous composition, with high thermal stability and high water expansion capacity, a surface with micro and macropores, and good adhesion of Wharton’s jelly mesenchymal stem cells (MSCWJ). We verified the absence of cytotoxicity and hemolytic activity, in addition, BB did not stimulate the activation of macrophages. Proving to be a safe material for direct cultivation and also for manufacturing of compounds used for in vivo applications.

## Introduction

Buritizeiro (*Mauritia flexuosa*) is one of the most valuable Amazonian heritage from ecological, social and economic points of view. It usually grows in permanently or periodically flooded areas along rivers, forests and savannah (Silva et al., 2009). The oil extracted from the fruit has a high quality chemical profile due to the high levels of carotenoids and tocopherols, being β-carotene and β-tocopherol the main of their classes and the monounsaturated fatty acids with oleic acid as the main constituents, which can be used as precursors for polyurethanes (PU) (Freitas et al., 2017; Cândido et al., 2015).

The use of vegetable oils in the production of polymeric materials has grown considering its biodegradability, renewability and structural arrangement susceptible of strategic chemical modifications, besides the low cost, for the synthesis (Spontón et al., 2013). In addition to the inclusion of polyethylene glycol (PEG), plasticizer and non-toxic, the polymer synthesis helps in improving the physical qualities of the material, increasing the pore density (Bhaskar et al., 2018; Chen et al., 2015; Scaffaro et al., 2016).

Synthetic polymers are once promising materials that have a wide range of applications, including tissue regeneration and tissue engineering (Gabriel et al., 2016; Pedersen et al., 2022). However, there is little availability of national biomaterials, which induces a subordination of external technology, consequently devaluing the national market (Rosa, 2017).

Mesenchymal stem cells (MSCs) are widely investigated for their repairing, anti-inflammatory and antioxidant properties. These characteristics are coupled to the secretion vesicles released by MSCs that contain several biomolecules with high mobility, providing a stable mechanism for biological signals (Barreto et al., 2017; Phelps et al., 2018). Thus, the use of porous matrices as a basis for the development and expansion of these cells has shown success, increasing its therapeutic efficacy (Carmagnola et al., 2018) and reproduction (Sousa et al., 2021).

This work aimed to analyze a Buriti oil based biomaterial, within its physicochemical properties, biocompatibility and cellular integration, with purpose to its use as a growth matrix for Goat Wharton’s jelly mesenchymal stem cells.

## Methods

### Synthesis of Buriti oil biomaterial

The biomaterials were produced from Buriti oil polymer (*Mauritia flexuosa*), at the Interdisciplinary Laboratory of Advanced Materials (LIMAV) of the Federal University of Piauí, Teresina-PI. Initially, the monoglyceride of Buriti oil was stirred at constant temperature, was added polyethylene glycol (PEG) and stirring was continued until a homogeneous mixture was formed. Then, the polymerizing agent, hexamethylene diisocyanate (HDI) (99%, Sigma-Aldrich) was added to the blend, the polymer was left for 24 hours at the initial reaction temperature to be finalized for use in this experiment, and thus, the biomaterials were cut at 10x10x3 mm, and autoclaved at 121 ºC (1 atm, relative pressure) for 15 minutes.

### Physical-chemical characterization of Buriti oil biomaterial

The characterization was performed using the following spectroscopy: X-ray diffraction (Drx) in a LABX-DRX 600, Shimadzu, Cu-Kα (λ = 1.5406Å) with 2θ in the range of 5º to 75º, with scanning rate of 2º min^-1^ and exposure time of 40 minutes; Fourier transform infrared spectroscopy using a Vertex 70 FTIR spectrograph from the manufacturer Brucker Optics with purge pump, wavelength between 400 cm^-1^ to 4000 cm^-1^ in transmittance modulus with 128 scans for each sample, made by KBR tablets; thermal analysis, using the Q 600 SDT, INSTRUMENTS, with a heating rate of 10 ºC/min, in the range of 25 to 600 ºC, N_2_ atmosphere, with a sample mass of approximately 10 mg; the swelling of the biomaterial was determined by the percentage of water absorption according to Pal and Pal (2006). The weights of the biomaterials (W1) were obtained, then immersed in PBS (pH 7.4) at 37 ºC for 24 hours, and soon after this period were again weighed (W2). The swelling (S) rate was calculated using the following:


$$S\left(\text{\%}\right)=\frac{W1-W2}{w1\;\times \;100}$$ (1)

The total porosity (P) of the biomaterials was determined using a liquid displacement method (Torres et al., 2013; Serra et al., 2015). After obtaining the SB (W1) weights, they were immersed in absolute ethanol (EtOH) for 48 hours, after which the biomaterials were weighed (W2). The porosity (P) was determined by the equation (%) = P**2**-P**1** **d*etanol*** × ***Vscaf fold*** **x100**. Detanol represents the density of ethanol and Vbiomaterial is the volume of wet biomaterial.

### Caprine stem cell and murine macrophage cultures

Goat Wharton’s jelly mesenchymal stem cells (MSCWJ), previously characterized and cryopreserved at the Integrated Nucleus of Morphology and Stem Cell Research (NUPCelt) of the Federal University of Piauí, were used. The cells had good plastic adherence, cell growth with well defined phases, plasticity capacity in osteogenic, adipogenic and chondrogenic lineages, expressed CD90 and CD105 markers and demonstrated absence of CD14 expression (Silva, 2016).

MSCWJ were thawed in a water bath at 37 ºC, double of the volume of DMEM/F-12 culture medium (12500062 Gibco®) was added. The cells were centrifuged at 300G for 10 minutes, the supernatant was discarded, and cultured using DMEM/F-12 medium supplemented with 20% fetal bovine serum (10437028 Gibco™), 1% penicillin and streptomycin [10,000 μg/mL] (15140122 Gibco™), 1% L-glutamine [2μM] (25030081 Gibco™) and 1% non-essential amino acids (11140050 Gibco™). Cell viability was determined by the Trypan blue method.

Murine macrophages were collected from the peritoneal cavities of 4-5week old male and female BALB/c mice from the Medicinal Plants Research Center (NPPM/CCS/UFPI), located at Teresina, PI, Brazil. The mice were maintained at a temperature of 24 ± 1 ºC and a 12 h light/dark cycle. All protocols were approved by the Animal Research Ethics Committee (CEUA/UFPI nº 00117/2015).

### Biointegration with MSCWJ

The adhesion of the MSCWJ in the BB was evaluated by scanning electron microscopy (SEM) and histological analysis. To that end, 1x10^5^ of the MSCWJ in fourth passage, were cultured in the biomaterial, after three days of culture three samples were fixed with 2.5% glutaraldehyde for 24 hours, and the remainder (three samples) were soaked in inclusion medium (Eadypath, Erviegas, Brazil), cooled to -40 ºC and sectioned in cryostat (HYRAX C 25, Zeiss, Germany) with a thickness of 5 μm and stained with eosin and hematoxylin. After the fixation, the samples destined to SEM were washed with PBS and dehydrated with slow water exchange using a series of dilutions of ethanol (30%, 55%, 70%, 88%, 96% and 100%) for 20 minutes at each concentration. After drying, the samples were deposited in carbon strips on aluminum supports and metalized with gold, the micrographs were collected from secondary electrons using a scanning electron microscope (QUANTA FEG 250). The pore dimensions, as they appear in the SEM images, were determined individually through the image processing using the ImageJ program

### Cytotoxicity determination

Cytotoxicity of BB was assessed using the MTT test. In a 24-well plate, 500 μL of supplemented DMEM/F-12 medium and about 1x10^5^ MSCWJ in fourth passage were added per well, in triplicate. They were then incubated at 37 ºC in 5% of CO_2_ for 24 h to allow cell adhesion. After this time, two washes with supplemented DMEM/F-12 medium were performed to remove cells that did not adhere. Subsequently, BB was added. Cells were then incubated for 24, 48 and 72 h. At the end of the incubation, 50 μL of MTT (M2003 Sigma-Aldrich) diluted in phosphate- buffered saline (PBS) was added at a final concentration of 5 mg/mL (10% of volume) and was incubated for an additional 4 h at 37 ºC in 5% CO_2_. The supernatant was then discarded, and 100 μL Dimethyl sulfoxide (DMSO: 99%- 276855 Sigma-Aldrich) was added to all wells. The plate was stirred for 30 min at room temperature to complete formazan dissolution. Finally, spectrophotometric reading was conducted at 550 nm in an ELISA plate reader. Supplemented DMEM/F-12 medium was used as control (100% viability).

### Hemolytic activity

The hemolytic activity was investigated by incubating the BB with a suspension of 5% red blood cells (caprine) for 1 h at 37 ºC in assay tubes. The reaction was slowed by adding 200 μL of PBS, and then the suspension was centrifuged at 300 g for 10 min. Cell lysis was then measured with an ELISA plate reader (540 nm). The absence of hemolysis (blank control) or total hemolysis (positive control) was determined by PBS or Milli-Q sterile water, respectively. The results were determined by the percentage of hemolysis compared to the positive control, the experiments were performed in triplicate.

### Phagocytosis test

Murine peritoneal macrophages were plated at a concentration of 2x10^5^ cells in 24-well culture plates and incubated with BB. After 48 h of incubation at 37 ºC in 5%CO_2_,10 μL of zymosan color solution was added at a final concentration of 0.02% and incubated for 30 min at 37 ºC. Afterwards, 100 μL of Baker's fixative (formaldehyde 4% v/v, sodium chloride 2% w/v, and calcium acetate 1% w/v in distilled water) was added to stop the phagocytosis process. 30 minutes later, the plate was washed with 0.9% saline in order to remove zymosan that was not phagocytized by the macrophages. The supernatant was removed and added to 100 μL of extraction solution. After solubilization in a Kline shaker, the absorbances were measured at 550 nm by using an ELISA plate reader. Supplemented RPMI medium was used as control (Grando et al., 2009).

### Nitric oxide (NO) production

For the nitric oxide (NO) induction test, peritoneal macrophages (2 × 10^5^ per well) were plated and incubated with the BB after 24 h of incubation at 37 ºC and 5% CO_2_. Cell supernatants were transferred to another 96-well plate for nitrite dosing. The standard curve was prepared with sodium nitrite diluted in Milli-Q water at 1, 5, 10, 25, 50, 75, 100, and 150 µM in the appropriate culture medium. At the different timepoints, the standard curve was determined with the same volume of Griess reagent (1% sulfanilamide in 10% H_3_PO_4_[v:v] in Milli-Q water, added in equal parts to 0.1% naphthylenediamine in Milli-Q water), and the absorbance was read on a BioTek plate reader (model ELx800) at 550 nm. Lipopolysaccharide (LPS) was used as a positive control.

### Statistical analysis

T-tests or analysis of variance ANOVA followed by the Tukey's test were performed using the GraphPad Prism version 7.0 program, taking the value of p <0.05 as the maximum level of statistical significance.

## Results

**Figure 1:**
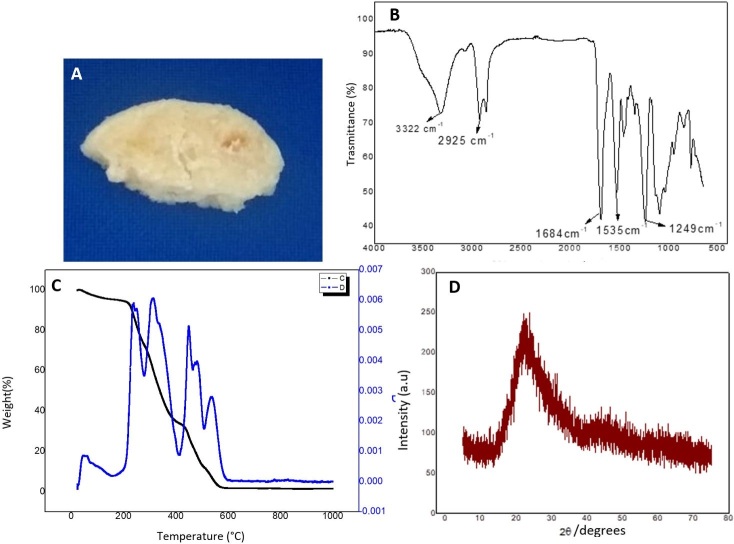
Physical-chemical characterization of buriti oil biomaterial. (A) Photograph of the Buriti oil Biomaterial section; (B) Infrared spectra with Fourier transform of Buriti oil biomaterial; (C) Thermogravimetric curve TG and DTG of thermal decomposition of Buriti oil polymer; (D) X-ray diffraction of Buriti oil biomaterial.

A light yellow color and elastic consistency biomaterial (Figure 1A) was obtained, with high resistance, demonstrating the efficiency of the materials used.

In the FTIR spectrum of the Buriti oil polymer (Figure 1B), the functional groups commonly found in polyurethanes are observed. The band around 3322 cm ^-1^ referring to the stretching of the OH and NH grouping; the peak around 2925 cm ^-1^, which is related to the C-H stretch; the band around 1684 cm ^-1^, which represents the C = O stretch of the ester group, as well as the band at 1535 cm ^-1^ of NH stretching and another band 1249 binds to the C-N-H group.

Figure 1C shows the TG and DTG curves of the BB. There are five stages of thermal degradation, the first event is related to structural components of the PU molecule with loss of mass 18.6% around 80 ºC; the second refers to the rupture of the urethane bonds from the HDI, used to obtain the PU, with loss of mass 10% at the temperature of 180 º C; in the third, fourth and fifth events, after 220 ºC.

**Figure 2:**
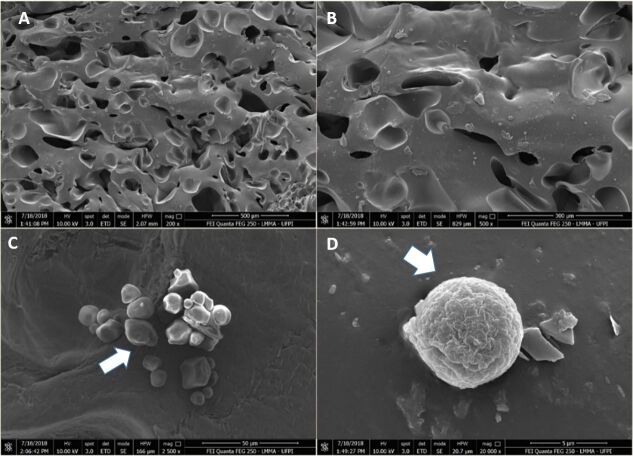
Scanning Electron Microscopy (SEM) of Buriti oil biomaterial cultivated with Wharton Jelly mesenchymal stem cells. (A) the porous structure of the biomaterials is shown; (B) rounded or fibroblastoid MSCWJ (C) and (D) SEM evidencing the MSCWJ with rounded morphology adhered to the biomaterial. Analysis after three days of cultivation.

Scanning Electron Microscopy (SEM) analysis showed that the biomaterial has in its structure micro and macro pores (Figure 2A), with dimensions ranging from 10 μm to 178 μm, average size of 78 μm.

The variation in the diameters of the polyurethane pores occurs, due to factors related to reagents and temperature, which can alter the characteristics of the materials. Figures 22C and 2D show the adhesion of MSCWJ cultivated with BB, the cells presented close contact with the matrix in several points, remaining intact, fact evidenced mainly by the presence of cells throughout the biomaterial. In this analysis, it was possible to confirm the adhesion of the cells in the biomaterial in question, but, it was only possible to visualize the cytoplasm and cell morphology.

**Figure 3:**
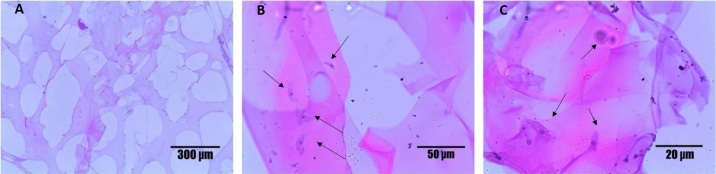
Photomicrograph of histological frozen section of the Buriti oil biomaterial cultured with Wharton’s Jelly Mesenchymal Stem Cells with Hematoxilin/Eosin staining. (A) The porous structure of the biomaterial; (B and C) the arrow points to rounded MSCWJ adhered in the biomaterial after three days of cultivation.

The photomicrographs (Figure 3) demonstrate that BB after 72 hours of culture had several adhered cells with fibroblastoid and rounded shapes. It stands out the presence of the stem cells in several layers of the biomaterial. The cellular nucleus was observed in Figure 3, with color purple and cytoplasm pink, thus the fibroblastoid and rounded morphologies are showed in Figure 3B and 3C.

**Figure 4:**
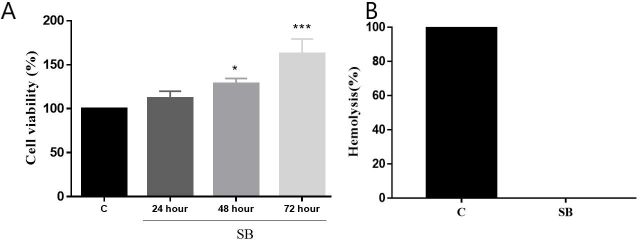
Viability of MSCWJ in Buriti oil polymer biomaterial. (A) Viability of MSCWJ in Buriti oil Biomaterial; (B) Hemolytic activity of the Buriti Biomaterial. Goat erythrocytes and mesenchymal stem cells of Wharton goat's jelly were incubated with BB. Each point represents mean ± standard error of the mean of three independent experiments performed in duplicate, with a confidence limit of 95%. Being p <0.05 (*), p<0.01 (***), (SB) Buriti oil polymer biomaterial, (C) Control.

These results demonstrate the effectiveness of BB as a functional framework for cell growth, highlighting the suitable size and interconnectivity of the pores, which facilitated cell adhesion and integration. In the assessment of cell viability, BB did not exhibit cytotoxic activity on MSCWJ and progressively increased cell viability (Figure 4A). Evaluations performed at 24, 48 and 72 hours increased cell metabolism by 12%, 28% (p <0.05) and 62% (p <0.001). It is suggested that the increase is due to the expansion of the cells in the biomaterial, which proves that BB promotes an conducive environment to the growth and multiplication of these cells. The biomaterial when in contact with blood cells does not present hemolytic activity (Figure 4B).

**Figure 5:**
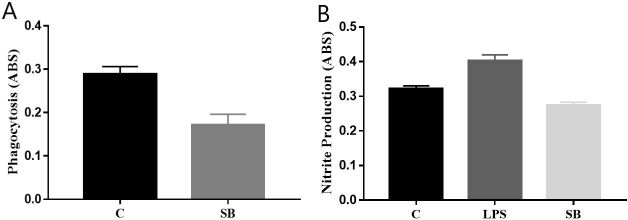
Activity of the Buriti Biomaterial on the activation of macrophages. (A) Analysis of the polyurethane phagocytosis of Buriti oil Biomaterial by macrophages; (B) Evaluation of the nitric oxide synthesis induced by the presence of the Buriti oil Biomaterial. Phagocytosis and nitric oxide synthesis were determined colorimetrically. The plot represents the mean ± standard error of the average of three independent experiments performed in triplicate. Being (SB) Buriti oil polymer biomaterial, (LPS) Lipopolysaccharide and (C) Control.

Regarding macrophage activation capacity, BB did not interfere significantly with the parameters evaluated. It can be observed in Figure 5A that there was a slight reduction in phagocytic capacity, as did the nitric oxide synthesis shown in Figure 5B.

## Discussion

The biomaterial synthesized from buriti oil allowed for both physical and chemical stability, serving as a viable matrix for mesenchymal stem cells. In this way, with regard to the absence of bands in the region 2000-2300 cm^-1^, confirms the participation of the isocyanate groups in the reaction, therefore, the isocyanate groups do not occur in the BB. The breakage of ester bonds from PEG occurs, with a loss of 71% of it’s, similarly to those provided by Cangemi (2006) in polyurethane analysis of vegetable oil.

According to the physical-chemical analysis presented by this study, it can be generally observed that porous materials have an unfavorable image, being often associated with contamination due to their difficult sterilization (Osorio et al., 2017). Thisstudy’s datademonstrate that BB has high thermal stability and can be sterilized by autoclaving process, reducing the chances of contamination for both in vitro culture and in vivo use in therapies. Figure 4 shows the x-ray diffractogram of the biomaterial. It is observed that it has a broad band, approximately between 2θ angles of 15º and 30º, with no sharp peaks, characteristic of non-crystalline polymers. These results are similar to those observed by Pollo et al. (2012).

Most of the polyurethanes are amorphous, lacking a periodic arrangement of atoms.. According to Araújo et al. (2011) in polyurethanes with high amount of rigid segments it is possible to observe peak 2θ near 12º. For Silva et al. (2010) polyurethanes with amorphous structures degrade faster than those with semicrystalline segments.

The BB presented porosity of approximately 67%, corroborating with the results found by Leite et al. (2016). According Paula and Trichês (2018), porosity is a fundamental characteristic, as it significantly influences cellular interactions, indicating a high presence of pores. The swelling rate of BB was observed to be approximately 50%. Wattanutchariya and Changkowchai (2014) suggest that greater and faster expandability and swelling of the biomaterial are more beneficial for the system.. Despite the hydrophobic character of the polyurethane matrix, there was a significant absorption of water, attributable to the porous structure of the matrix that permits water diffusion into its interior.

According to Yan et al. (2015), the presence of pores less than 20 μm diameter is useful for activating nutrients and metabolites, and pores with a diameter greater than 100 μm, are necessary for the proliferation and cell migration. These characteristics resemble those found by Bhaskar et al. (2018) in the analysis of polyurethanes with addition of PEG. Although, Bellincanta et al. (2011), obtained pore sizes varying from 5 to 7 μm of their polyurethanes, while, Macedo et al. (2017) reported diameters from 50 to 350 μm.

Results on cell viability and absence of cytotoxic activity by MSCGW in this research corroborate with Gabriel et al. (2016). Both works, using polyurethanes, showed no signs of cytotoxicity, however, the BB increased the viability of the cells, functioning as a matrix for expansion of the cells. The absence of hemolytic activity of the biomaterial aligns with findings described by Jaganathan and Mani (2018) and Yuan et al. (2008). Peng et al. (2018) demonstrated hemolytic activity in commercial polyurethanes, Tecoflex® whereas the BB presented a satisfactory result, with no hemolytic activity.

According to the results of the macrophage activation capacity assay, it is possible to infer that BB does not activate macrophages due to the biocompatibility of biomaterial with cells, which is a desirable feature in polymers and solid devices intended for biological applications. In vitro cell assays are valuable for mimicking tissue responses and are widely employed in the discovery of new materials (Costa Victal et al., 2015).

## Conclusion

The biomaterial, formed by combining polyurethane of the Buriti oil with polyethylene glycol, exhibits an amorphous structural arrangement and containsmicro and macropores. Additionally, it displays high thermal resistance and does not show any toxic effects onGoat Wharton’s jelly mesenchymal stem cells. Likewise, it does not induce alterations on macrophages, highlighting its biocompatible properties. Theof Buriti oil and PEG polymer shows significant potential for clinical applicationas it demonstrates to be a safe material for direct cultivation and for the manufacture of compounds used for in vivo applications.

## Abbreviations

MSCWJ, Wharton Jelly Stem Cell, PEG, polyethylene glycol; FTIR, spectroscopy Infrared region; TG, Thermogravimetric Analysis; SEM, scanning electron microscopy; MSC, mesenchymal stem cells; BB, biomaterial of Buriti oil and polyethylene glycol.
